# First clinical assessment of a prototype assay to detect the enzymatic activity of β-lactamase as a marker for pulmonary tuberculosis

**DOI:** 10.1016/j.diagmicrobio.2020.115026

**Published:** 2020-06

**Authors:** Pamela Nabeta, Pratibha Seshadri, Joshua Havumaki, Silindile Mbhele, Layla Hendricks, Mark D. Perkins, Mark P. Nicol, Claudia M. Denkinger

**Affiliations:** a FIND, Chemin des Mines 9, 1202, Geneva, Switzerland; b Division of Infectious Diseases, Beth Israel Deaconess Medical Center, 330 Brookline Ave, 02215, Boston, USA; c Division of Medical Microbiology and Institute for Infectious Diseases and Molecular Medicine, University of Cape Town, Anzio Rd, Observatory, Cape Town, 7925, South Africa and National Health Laboratory Service, South Africa; d School of Biomedical Sciences, University of Western Australia, Hackett Drive, Crawley, Perth, Australia 6009; e Division of Tropical Medicine, Center of Infectious Diseases, University Hospital of Heidelberg, Im Neuenheimer Feld 324, 69120, Heidelberg, Germany

**Keywords:** Tuberculosis, Diagnosis, TB REaD™, β-Lactamase

## Abstract

The objective was to evaluate the sensitivity and specificity of a novel prototype test, TB REaD™, a reporter enzyme fluorescence–based assay, for pulmonary tuberculosis and to determine the optimal threshold for test positivity. This blinded, prospective study enrolled 250 patients, of which 23.2% were *Mycobacterium tuberculosis* complex (MTB) culture-positive. At the manufacturer-set threshold, sensitivity of the assay was 93.1% (95% confidence interval [CI] 83.3–98.1) and specificity was 8.9% (95% CI 5.2–13.8). The highest accuracy was seen at a higher threshold: sensitivity 58.6% (95% CI 44.9–71.4), specificity 59.4% (95% CI 52.1%–66.4%), with sensitivity by smear status being 40.0% (95% CI 21.1–61.3) for smear-negative and 72.7% (95% CI 54.5–86.7) for smear-positive. This study demonstrated limited accuracy of the TB REaD™ prototype for detection of pulmonary TB. Further improvements are necessary, potentially exploring probes that are more specific to MTB.

## 1 Introduction

Tuberculosis (TB) is causing tremendous suffering worldwide and has surpassed HIV/AIDS as the world's leading single infectious cause of death. The World Health Organization (WHO) estimates that, in 2017, 10 million people became ill with TB globally. Approximately 1.3 million HIV-negative people and 300,000 HIV-positive people died from TB (World Health Organization, 2018). Accurate and rapid TB diagnosis is key to the control of this global epidemic, and particularly, rapid and simple diagnostics that reach patients in lower levels of care are needed (World Health Organization, 2014). Sputum smear microscopy remains the mainstay for TB diagnosis as, to date, novel molecular tests cannot be implemented in most microscopy centers where the majority of patients present to care (Albert et al., 2016). Biomarker-based tests that rely on antibody, antigen, or enzymatic detection in sputum or more easily accessible samples such as blood, urine, or stool are more likely to be suitable for implementation at the lowest level of health care systems (World Health Organization, 2014). Various biomarkers have been evaluated for the detection of *Mycobacterium tuberculosis* complex (MTB) (Wallis et al., 2013). One promising biomarker for detection of TB in sputum, with potential to be suitable for implementation at low levels of care (e.g., peripheral health centers), is the mycobacterial β-lactamase enzyme.

Some mycobacteria are known to produce β-lactamases and are intrinsically resistant to many β-lactam antibiotics (Kwon et al., 1995). MTB β-lactamase is coded by the *blaC* gene (Wang et al., 2006). In 2004, cell-permeable near infrared fluorogenic substrates, suitable for imaging of mammalian cells, were first developed (Weissleder, 2001; Xing et al., 2005).

Building on this knowledge, in 2010, a reporter enzyme fluorescence (REF)–based test using a near-infrared fluorogenic substrate (CNIR5) of naturally occurring mycobacterial β-lactamase, which allowed for real-time imaging and quantification of mycobacteria in mice was first described (Kong and Cirillo, 2010). The test allowed for identification of 10^4^ colony-forming units (cfu) in live mice with the potential for tracking therapeutic efficacy through the loss of REF signal posttreatment (Kong and Cirillo, 2010). This was followed by the development of a green fluorescent probe, CDG-OMe, based on a chemically modified cephalosporin, which enabled increased specificity and imaging of low quantities of *Bacillus* Calmette-Guérin and MTB (as low as 10 cfu) in human sputum in early proof-of-principle studies (Xie et al., 2012).

The fluorogenic probe used on the TB REaD assay, CDG-3, has demonstrated a stronger fluorescent signal as well as increased specificity to mycobacterial *blaC* compared to CDG-OMe in a study by Cheng et al. In this study utilizing fresh sputum samples from 50 subjects with presumptive TB, CDG-3 detection demonstrated 100% sensitivity in smear-positive, culture-positive cases; 80% sensitivity in smear-negative, culture-positive cases; and 73% specificity (Cheng et al., 2014).

This probe was the basis for a prototype assay for detection of *M. tuberculosis* in sputum developed by Global BioDiagnostics Corp. (GBDbio, Temple, TX), the GBDbio TB REaD™ assay (TB REaD), which was intended to be suitable for point-of-care testing at level one. Here we determine the accuracy of this assay in comparison with sputum smear microscopy and Xpert MTB/RIF using mycobacterial culture as the reference standard.

## 2 Methods

### 2.1 Study design

This blinded, prospective, laboratory-based study was performed by the Foundation for Innovative New Diagnostics (FIND, Geneva, Switzerland) in partnership with the Division of Medical Microbiology at the University of Cape Town, South Africa. Individuals attending a primary health care clinic who were aged 18 years or above, with persistent cough (≥2 weeks) and at least 1 other symptom of pulmonary TB (fever, night sweats, malaise, recent weight loss, contact with active case, hemoptysis, chest pain, or loss of appetite), and able to provide informed consent were included in the study. Individuals with signs compatible with only extrapulmonary disease and those having received more than 2 doses of anti-TB therapy prior to enrolment were excluded. Participants were asked to provide 2 spot sputum samples within 2 days for testing, and HIV testing was offered.

### 2.2 Reference standard

**Fig. 1:**
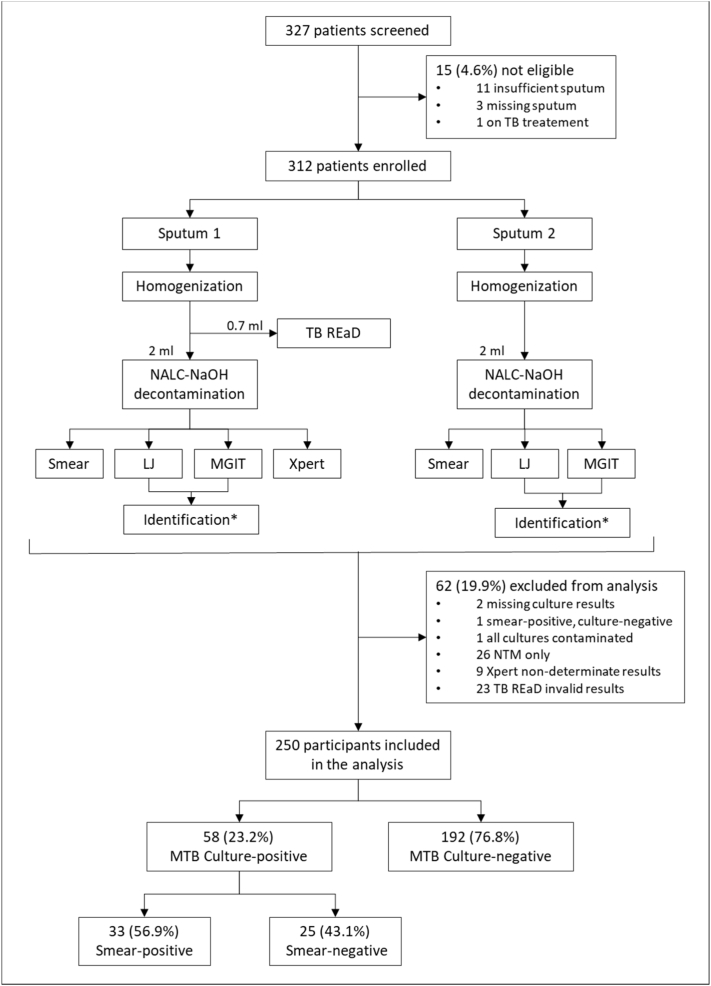
Study flow diagram. *MTB identification from first positive culture. Abbreviations: LJ = Löwenstein-Jensen; MGIT = mycobacteria growth indicator tube; NTM = nontuberculous mycobacteria; MTB = *Mycobacterium tuberculosis* complex.

Sputum characterization was performed at a quality-assured TB reference laboratory, as per the study flowchart shown in Fig. 1. Both sputum samples (minimum volume 2.5 mL) were homogenized and vortexed with sterile glass beads before processing. This was done to enable a uniform sample to be split across tests without advantaging one or the other. The first sputum sample was split into 2 aliquots: 1 for TB REaD and 1 for the reference standard. The latter underwent standard *N*-acetyl-l-cysteine and sodium hydroxide decontamination, and the resuspended pellet was used to perform LED fluorescent smear microscopy, liquid culture using mycobacteria growth indicator tube with a BACTEC 960 instrument (BD Microbiology Systems, Sparks, MD), and solid culture with Löwenstein-Jensen medium. In addition, Xpert® MTB/RIF (Cepheid, Sunnyvale, CA [Xpert]) was performed from the resuspended pellet of the first sputum sample. The second sputum sample was subjected to decontamination followed by LED fluorescent smear microscopy, and liquid and solid culture. MTB complex identification was performed using Line Probe Assay (Hain, Nehren, Germany) from the first positive culture on each sputum sample.

### 2.3 TB REaD training

The study was preceded by a training phase where 2 laboratory technicians and a laboratory supervisor were trained on the TB REaD procedure that was developed by GBDbio (See Fig. 2.). Hands-on training included testing of approximately 25 fresh, residual sputum samples measured with the TB REaD fluorescent reader. Operational performance was monitored by FIND using a pre-established checklist.

**Fig. 2:**
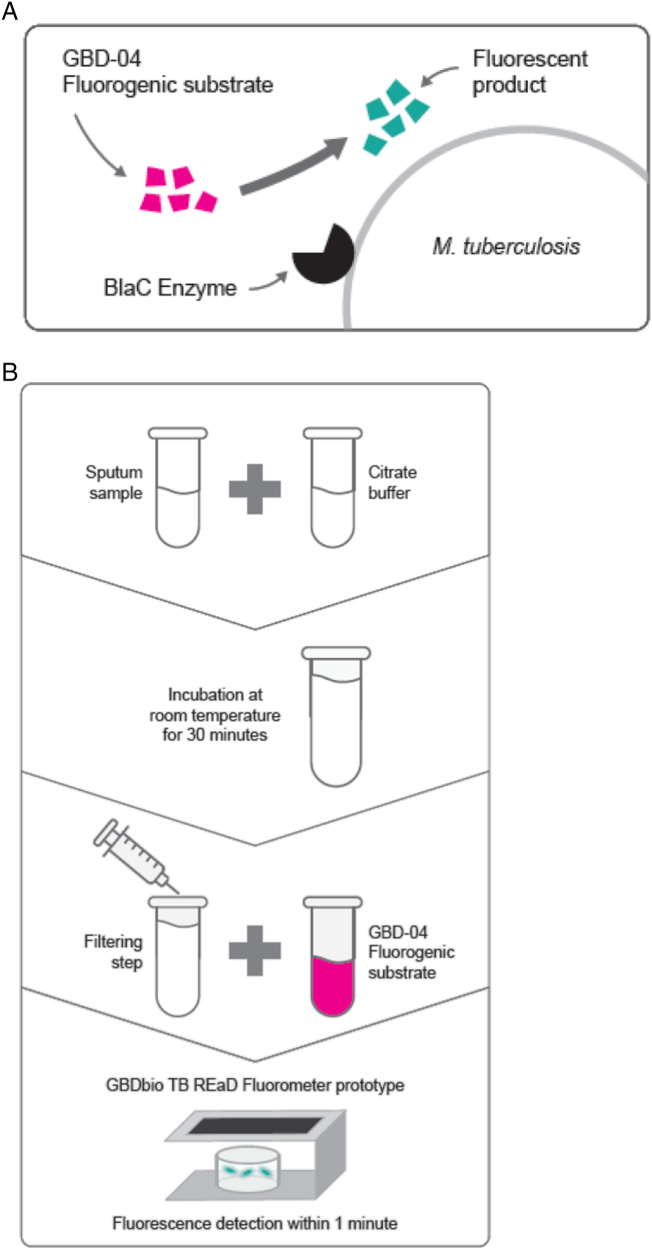
(A) TB REaD assay principle. (B) Scheme showing the workflow of the prototype assay, TB REaD, for detection of *M. tuberculosis* on sputum samples.

### 2.4 TB REaD clinical sample analysis

TB REaD was done directly from the first sample in real time at the research laboratory of Mark Nicol at the University of Cape Town. A total of 700 μL of homogenized, otherwise raw sputum was used as input volume for TB REaD as defined by the test developer. The sputum was mixed with citrate buffer and incubated for 30 min. The sample mix was then filtered (using a proprietary filter of the company), and after addition of the GBD-04 fluorogenic substrate, the prepared material was directly read using the GBDbio TB REaD fluorometer (the prototype instrument) within 1 min (Fig. 2).

**Fig. 2:**
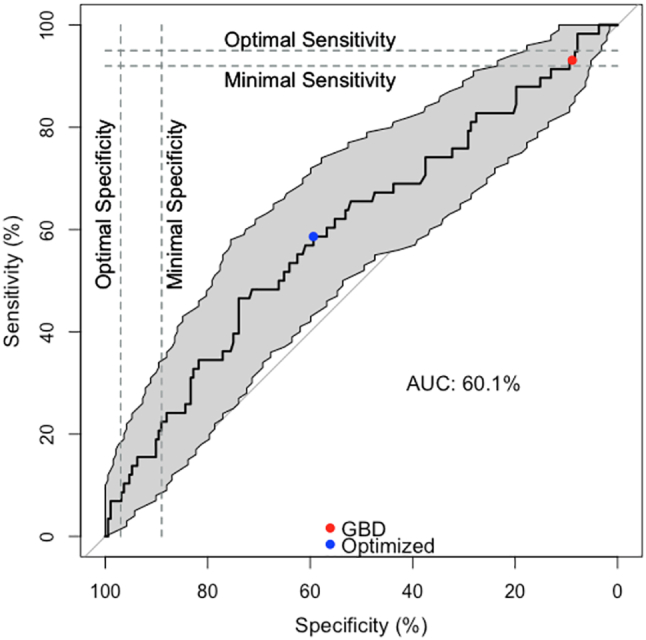
TB REaD performance against culture for different thresholds with 95% CIs calculated using the Youden index. The dashed lines indicate the minimal and optimal sensitivity and specificity as defined in the WHO target product profile. The gray area indicates the confidence interval. GBD = manufacturer-set threshold; Optimized = threshold to reach best performance; AUC = area under the curve.

Operators of the TB REaD were blinded to the patients' microbiological results and clinical diagnosis. Quality assurance included the use of external controls (positive and negative). Date and time of sample collection and date and time of TB REaD assay testing were recorded. Sputum was either tested on the same day or refrigerated at 4 °C for testing the next day.

### 2.5 Statistical analysis

Data were captured through a dedicated, online, password-protected double data entry system. A culture-positive case was defined as a participant with at least 1 culture testing positive for MTB on either of the 2 samples, whereas a culture-negative case was defined as a participant with all negative cultures for MTB (out of 4) or at least 2 negative cultures for MTB and 2 contaminated cultures (i.e., no positive culture). Study participants were considered smear-positive, culture-positive if they had at least 1 positive smear (inclusive of scanty positive smears). Participants with unclear microbiological diagnosis were excluded from the analysis, including missing smear and/or culture, smear-positive but culture-negative, contamination of at least 3 out of 4 inoculated cultures, growth of nontuberculous mycobacteria only, as well as nondeterminate Xpert or invalid TB REaD results.

The primary end point was to determine the accuracy of the current TB REaD prototype at the threshold provided by the test manufacturer using culture as the reference standard. Secondary end points included the assessment of the performance of TB REaD i) at the best performing cutoff value and ii) in comparison to diagnostic sensitivity and specificity requirements according to the target product profile characteristics of a rapid sputum-based test for detecting TB at the microscopy center level approved by WHO (World Health Organization, 2014).

This was the first independent clinical study to assess the performance of TB REaD. Based on 20–25% TB prevalence, a target recruitment of 300 participants was estimated to provide 60–75 culture-positive cases to allow initial point estimates for sensitivity and specificity with acceptable confidence intervals (CIs). We performed all the analyses with Stata, Version 12 (Stata Corp, College Station, TX). The reporting followed the Standards for the Reporting Diagnostic Accuracy (Bossuyt et al., 2015).

The study protocol was approved by the Human research Ethics Committee of the University of Cape Town.

## 3 Results

We screened 327 patients, of which 312 met the study inclusion criteria (Fig. 1). Of these, 250 patients were included in the analysis. A total of 58 were culture-positive TB (23.2%), of which 33 were smear-positive (56.9%).

The mean age of participants was 39.0 years, 46% of participants were male, 44% had a prior history of TB, and 62% were patients living with HIV. There was no difference in these characteristics between culture-positive and culture-negative groups (Table 1).

**Table 1:** Demographic characteristics.

	All participants (*N* = 250)	All culture-positive (*N* = 58)	Culture-negative (*N* = 192)
Mean age, y (SD)	39.0 (11)	38.7 (12.7)	39.1 (10.4)
Male (*n*/*N*)	46.4% (116/250)	51.7% (30/58)	44.8% (86/192)
History of TB (*n*/*N*)	44.4% (111/250)	39.7% (23/58)	45.8% (88/192)
HIV positive (*n*/*N*)	62.4% (156/250)	55.2% (32/58)	64.6% (124/192)

SD = standard deviation; *n* = number of participants meeting criterion; *N* = total participants.

At the manufacturer-set threshold (2230.0 arbitrary units [au]), assay sensitivity was 93.1% (95% CI 83.3–98.1); however, specificity was only 8.9% (95% CI 5.2–13.8) (Table 2). The highest accuracy of TB REaD was seen at a higher threshold (3198.5 au; “optimized threshold”), with a sensitivity of 58.6% (95% CI 44.9–71.4) and specificity of 59.4% (95% CI 52.1%–66.4%). Sensitivity correlated with smear status. Among smear-positive samples, culture-positive sensitivity was 72.7% (95% CI 54.5–86.7) at this optimized threshold. For smear-negative, culture-positive participants, sensitivity at the same threshold was 40.0% (95% CI 21.1–61.3) (Table 3).

**Table 2:** Sensitivity and specificity of TB REaD at the manufacturer-set threshold and optimized threshold in comparison to Xpert MTB/RIF with culture as reference standard.

Assay		Sensitivity (95% CI) [TP/(TP + FN)]	Specificity (95% CI) [TN/(TN + FP)]
TB REaD	GBD-set threshold (2230 au)	93.1% (83.3–98.1) [54/58]	8.9% (5.2–13.8) [17/192]
	Optimized threshold (3198.5 au)	58.6% (44.9–71.4) [34/58]	59.4% (52.1–66.4) [114/192]
Xpert MTB/RIF		84.5% (72.6–92.7) [49/58]	98.4% (95.5–99.7) [189/192]

GBD = manufacturer; au = arbitrary units; CI = confidence interval; TP = true positive; FN = false negative; TN = true negative; FP = false positive.

**Table 3:** Performance of TB REaD in comparison to Xpert MTB/RIF by smear and HIV status.

			Culture positive (58)		Culture positive (58)	
			Smear status		HIV status	
			+ (33)	− (25)	+ (32)	− (26)
TB REaD	GBD-set threshold (2230 au)	+	31	23	31	23
		−	2	2	1	3
	Optimized threshold (3198.5 au)	+	24	10	15	19
		−	9	15	17	7
Xpert MTB/RIF		+	32	17	26	23
		−	1	8	6	3

GBD = manufacturer; au = arbitrary units.

When looking at the sensitivity of TB REaD by HIV status at the manufacturer-set threshold, the assay performed slightly better among HIV-positive than among HIV-negative individuals 96.9% (95% CI 83.8–99.9) and 88.5% (69.8–97.6), respectively, with specificity at 8.9% (95% CI 5.2–13.8). At an optimized threshold, sensitivity was slightly higher among HIV-negative cases; however, the CIs were overlapping (Table 3).

To account for potential sample-to-sample variability, we compared the sensitivity of TB REaD using only 2 cultures on the same sample on which TB REaD was performed as reference standard (Appendix), and the sensitivity was maintained for TB REaD. The receiver operating characteristic curve (Fig. 2) visualizes the sensitivity and specificity with the changing TB REaD threshold, including the 95% CI. Additionally, the graph shows the comparison to the diagnostic sensitivity and specificity point estimates according to the WHO-approved target product profile characteristics. The total area under the curve was 60.1%.

## 4 Discussion

In this cross-sectional diagnostic accuracy study, we assessed sputum specimens collected from adults with presumed TB for the detection of pulmonary TB. Sputum specimens were tested with a novel assay, the TB REaD, representing its first clinical study. The TB REaD assay employs the use of a unique fluorogenic substrate to identify β-lactamase produced by MTB as a marker for pulmonary TB.

Overall, the assay's performance was limited and did not come close to the minimal targets put forward by WHO for a sputum-based test to replace smear microscopy (World Health Organization, 2014). The sensitivity of TB REaD at an optimized threshold for highest accuracy on this sample set was comparable to smear microscopy in all sputum samples combined (58.6% versus 56.9%) but inferior in sputum smear-positive samples (72.7%). Furthermore, specificity (59.4%) was substantially inferior to what is typically observed with smear microscopy. The performance of TB REaD was also suboptimal in comparison to Xpert in terms of sensitivity (58.6% versus 84.5% overall, 40% versus 68% among smear-negative, culture-positives) and specificity (59.4% versus 98.4%). Overall, we were unable to replicate the results published by Cheng on the CDG-3 probe with the assay utilizing this probe in our study (Cheng et al., 2014).

We recognize the limitations of our study. This was an early feasibility study with limited sample size. We were unable to characterize the sputa of patients further to identify bacteria that are possibly responsible for the cross-reactivity. An exclusion of nontuberculous mycobacteria per protocol might have modified the performance results, likely to the benefit of the index test.

The results highlight that, at the present configuration, the test would not have a role in the diagnostic cascade. The most likely reason for the insufficient performance of the test is the limited specificity of the β-lactamase, which is an enzyme present across a wide range of bacteria. β-Lactamase activity was observed to wane rapidly over time in analytical studies by the test developer, and any delays during sample processing may affect the sensitivity of the test. However, in our study, the time to collection/testing and the transport conditions were controlled and thus unlikely to have contributed to the observed results. Nevertheless, this also raises concerns about the challenges for implementation of the test, as time-sensitive steps may be less controlled in routine laboratories with high workload.

Should the performance of the assay be substantially improved through more specific probes, the assay could have a potential application given its ease of use and its rapid turnover of clinical samples.

In summary, this first accuracy study of the prototype TB REaD assay done on fresh sputum samples demonstrated limited sensitivity and specificity. Substantial assay optimization would be required using more specific fluorogenic substrates and better sputum processing methods for which variability in time to processing is less critical to improve the performance of this test.

## References

[bb0005] Albert H., Nathavitharana R.R., Isaacs C., Pai M., Denkinger C.M., Boehme C.C. (2016). Development, roll-out and impact of Xpert MTB/RIF for tuberculosis: what lessons have we learnt and how can we do better?. Eur Respir J.

[bb0010] Bossuyt P.M., Reitsma J.B., Bruns D.E., Gatsonis C.A., Glasziou P.P., Irwig L. (2015). STARD 2015: an updated list of essential items for reporting diagnostic accuracy studies. BMJ.

[bb0015] Cheng Y., Xie H., Sule P., Hassounah H., Graviss E.A., Kong Y. (2014). Fluorogenic probes with substitutions at the 2 and 7 positions of cephalosporin are highly BlaC-specific for rapid mycobacterium tuberculosis detection. Angew Chem Int Ed.

[bb0020] Kong Y., Cirillo J.D. (2010). Reporter enzyme fluorescence (REF) imaging and quantification of tuberculosis in live animals. Virulence.

[bb0025] Kwon H.H., Tomioka H., Saito H. (1995). Distribution and characterization of beta-lactamases of mycobacteria and related organisms. Tuber Lung Dis.

[bb0030] Wallis R.S., Kim P., Cole S., Hanna D., Andrade B.B., Maeurer M. (2013). Tuberculosis biomarkers discovery: developments, needs. and challenges Lancet Infect Dis.

[bb0035] Wang F., Cassidy C., Sacchettini J.C. (2006). Crystal structure and activity studies of the mycobacterium tuberculosis beta-lactamase reveal its critical role in resistance to beta-lactam antibiotics. Antimicrob Agents Chemother.

[bb0040] Weissleder R. (2001). A clearer vision for in vivo imaging. Nat Biotechnol.

[bb0045] World Health Organization (2014). High-priority target product profiles for new tuberculosis diagnostics: Report of a consensus meeting. Programme GT, editor.

[bb0050] World Health Organization (2018). Global tuberculosis control: WHO report 2018.

[bb0055] Xie H., Mire J., Kong Y., Chang M., Hassounah H.A., Thornton C.N. (2012). Rapid point-of-care detection of the tuberculosis pathogen using a BlaC-specific fluorogenic probe. Nat Chem.

[bb0060] Xing B., Khanamiryan A., Rao J. (2005). Cell-permeable near-infrared fluorogenic substrates for imaging beta-lactamase activity. J Am Chem Soc.

